# Immune checkpoint inhibitors continuation beyond progression as second-line treatment for extensive-stage small-cell lung cancer: a real-world, multicenter analysis

**DOI:** 10.3389/fimmu.2025.1666373

**Published:** 2025-11-11

**Authors:** Jiao Zhang, Rui Zi, Hai-xia Zhang, Ye Lv, Lu-jun Zhao, Yan Wang

**Affiliations:** 1 The Third Department of Medical Oncology, General Hospital of Ningxia Medical University, Yinchuan, Ningxia, China; 2 The First Department of Medical Oncology, General Hospital of Ningxia Medical University, Yinchuan, Ningxia, China; 3 Department of Radiation Oncology, Tianjin Medical University Cancer Institute and Hospital, National Clinical Research Center for Cancer, Tianjin Key Laboratory of Cancer Prevention and Therapy, Tianjin’s Clinical Research Center for Cancer, Tianjin, China

**Keywords:** extensive-stage small cell lung cancer, chemoimmunotherapy, second-line therapy, survival, prognosis

## Abstract

**Background:**

Optimal management of extensive-stage small-cell lung cancer (ES-SCLC) following progression on first-line (1L) chemoimmunotherapy remains undefined. This study aimed to evaluate the efficacy of immune checkpoint inhibitors (ICIs) continuation in a second-line (2L) treatment setting.

**Methods:**

A total of 211 ES-SCLC patients with disease progression after 1L chemoimmunotherapy were analyzed retrospectively after stratifying them into ICIs continuation (n = 118) and ICIs discontinuation (n = 93) cohorts. The primary endpoint was 2L overall survival (2L-OS), and the secondary endpoints included 2L progression-free survival (2L-PFS), objective response rate (2L-ORR), disease control rate (2L-DCR), and safety. Propensity score matching (PSM, 1:1) ensured balanced baseline characteristics. Survival analyses were conducted based on Kaplan-Meier curves. Univariate and multivariate Cox regression analyses were performed to identify the factors associated with 2L-PFS and 2L-OS.

**Results:**

ICIs continuation significantly improved 2L-OS (8.66 vs 7.90 months; P = 0.016) and 2L-PFS (3.92 vs. 2.15 months; P < 0.001). The benefits of ICIs continuation persisted after PSM (2L-OS: 10.31 vs. 8.95 months, P = 0.027; 2L-PFS: 4.22 vs.2.12 months, P < 0.001). In addition, the ICIs continuation group demonstrated superior tumor response (2L-ORR: 28.8% vs. 11.8%, P = 0.003; 2L-DCR: 65.3% vs. 44.1%, P = 0.002), which remained significant post-PSM. Treatment-related adverse events (AEs) were comparable between the groups, while immune-related AEs were predominantly low grade in the ICIs continuation group. Multivariate analysis revealed that baseline liver metastasis and 1L-PFS were independent risk factors for 2L-PFS and 2L-OS, whereas overweight (BMI 25.0-29.9) was an independent prognostic factor for 2L-OS. The exploratory analysis conducted for the ICIs continuation cohort revealed no significant difference in patient survival between the continuing ICIs treatment group and switching ICIs treatment group (2L-OS: P = 0.668; 2L-PFS: P = 0.346).

**Conclusion:**

In patients with ES-SCLC who exhibit disease progression after 1L chemoimmunotherapy, continuation of ICIs significantly improves survival and tumor response while achieving a manageable safety profile. Therefore, ICIs continuation may be considered a viable strategy in 2L settings.

## 1 Introduction

Lung cancer is the leading cause of cancer-related mortality worldwide (1). However, the epidemiological trends of this disease vary significantly between China and the United States. A comparative study revealed that while the U.S. witnessed declining incidence and mortality rates for both sexes from 2000 to 2018, China faced a contrasting picture with a rising incidence trend among females, despite decreasing mortality in both sexes (2). These divergent patterns highlight differing stages of epidemiological transition and underscore the urgent need for tailored prevention strategies in China, particularly in tobacco control. Small cell lung cancer (SCLC), the most aggressive lung cancer subtype strongly associated with tobacco exposure (3). SCLC accounts for about 15% of all cases of lung cancers reported worldwide and is characterized by rapid proliferation, early metastasis, and a poor prognosis (4). About 70% of the SCLC patients present with extensive-stage disease (ES-SCLC) at initial diagnosis (5), and these cases represent a particularly challenging subset of this disease. In prior studies, first-line (1L) treatment with platinum-etoposide chemotherapy was reported to exhibit high efficacy in ES-SCLC patients, with response rates ranging from 60% to 65% (6). However, despite this initial responsiveness, most eventually developed resistance and experienced disease progression within one year, and the 5-year overall survival (OS) rate remained <5% (7).

The emergence of immune checkpoint inhibitors (ICIs) in recent years has significantly altered the landscape of ES-SCLC treatment. The integration of ICIs, specifically the PD-L1 inhibitors such as atezolizumab and durvalumab, with platinum-based chemotherapy has become the standard 1L treatment currently, and has led to significant improvements in OS (8, 9). Recent phase III trials have further confirmed these benefits with the use of novel ICIs such as adebrelimab (median OS 15.3 months, hazard ratio (HR) 0.72) and serplulimab (median OS 15.4 months, HR 0.63) (10, 11). These advancements have extended survival and also improved the quality of life for many patients with ES-SCLC. However, nearly all patients are expected to eventually experience disease progression due to the intrinsic biological aggressiveness of SCLC. The absence of validated biomarkers further complicates the stratification of patients (12). Unfortunately, the therapeutic options for treating disease progression remain limited. In the chemotherapy era, guidelines recommend second-line (2L) chemotherapy or clinical trial enrollment, although these approaches also demonstrate suboptimal efficacy, with a median progression-free survival (PFS) of just 2–3 months. In this context, the mainstream 2L treatment strategy for SCLC has historically relied on chemotherapeutic agents. Topotecan, a topoisomerase inhibitor, has been the long-standing standard of care, albeit with modest efficacy and significant toxicity, particularly severe myelosuppression which often limits its use (13). More recently, lurbinectedin has emerged as an approved option in some regions, showing improved response rates, but its accessibility is hampered by high cost and limited reimbursement, especially in China (14). A significant breakthrough has been the approval of tarlatamab, a bispecific T-cell engager (BiTE) therapy targeting DLL3 on SCLC cells, which has demonstrated durable responses and a survival benefit (15). However, this promising agent is not yet approved in China, leaving a substantial gap for patients. Additionally, rechallenge with first-line platinum-based chemotherapy may be considered for patients with a treatment-free interval of more than 6 months (16). Collectively, the challenging trade-offs in the second-line SCLC landscape—between efficacy, toxicity, and accessibility—culminate in a persistent and critical unmet medical need.

Critically, the optimal regimens after 1L chemoimmunotherapy remain undefined to date, and whether ICIs continuation will continue to provide survival benefits for patients with 1L chemoimmunotherapy resistance remains unclear so far. This gap in understanding has driven the exploration of novel therapeutic strategies, including the continuation of ICIs in 2L treatment settings. In cancer types other than SCLC, such as non-small cell lung cancer (NSCLC) and melanoma, ICIs continuation beyond disease progression in 2L treatment settings demonstrates promising results (17, 18); for example, patients with NSCLC who continued ICIs after disease progression beyond 1L reportedly experienced durable responses and improved survival. Similarly, in some patients with melanoma, the continuation of ICIs was associated with prolonged disease control. These findings suggest that the immune response elicited by ICIs may be sustained over time, and this provides a rationale for the continuation of ICIs use in 2L treatment settings. However, the applicability of these findings to ES-SCLC remains to be elucidated because of the distinct biology and immunological profile of SCLC. Recent studies have explored the potential benefits of ICIs continuation in the 2L treatment of ES-SCLC patients (19, 20). The rationale behind this approach is based on the observation that some patients may derive prolonged benefit from ICIs, even after disease progression is noted initially in these patients. The immune response to cancer is a complex and dynamic phenomenon, and ICIs continuation may help maintain immune control over the disease. However, previous studies have reported inconsistent results in terms of the efficacy of ICIs continuation in 2L treatment settings, with some studies reporting significant improvements in OS and PFS (19–23), while others have reported limited benefits (24, 25).

Since conventional 2L treatments in ES-SCLC have exhibited limited efficacy, the benefits of ICIs continuation observed in other cancers, and the inconsistent findings regarding 2L ICIs in ES-SCLC, exploring the role of ICIs continuation in the 2L treatment of ES-SCLC is of paramount importance. Therefore, our study aimed to evaluate the efficacy of ICIs continuation after disease progression on 1L chemoimmunotherapy.

## 2 Materials and methods

### 2.1 Patients

Patients with ES-SCLC who underwent 2L treatment after disease progression noted in 1L chemoimmunotherapy were analyzed retrospectively in this study. Patients who underwent treatment at Tianjin Medical University Cancer Institute and Hospital, General Hospital of Ningxia Medical University, and the Affiliated Hospital of Inner Mongolia Medical University between March 2019 and December 2023 were enrolled as the study population in this study. The inclusion criteria for the study were as follows: (1) Age ≥ 18 years. (2) Pathological or cytological confirmation of SCLC. (3) Extensive-stage disease at the initial diagnosis, according to the definition of the Veterans Administration Lung Study Group (VALG) staging system. (4) Disease progression noted after 1L of chemoimmunotherapy. (5) Availability of all (complete) clinical and medical records. The exclusion criteria were as follows: (1) Histopathological revelation of SCLC combined with other cellular components (e.g., adenocarcinoma or large cell carcinoma). (2) History of other concurrent malignancies.

The last follow-up date for the study population was March 1, 2025. After enrolment, the patients were stratified into the ICIs continuation group and the ICIs discontinuation group based on whether ICIs were continued in 2L treatment.

### 2.2 Data collection

Baseline clinical characteristics at diagnosis, including gender, age, smoking status, Eastern Cooperative Oncology Group (ECOG) performance status (PS), Body Mass Index (BMI), and metastatic sites, were retrieved from the electronic medical records of patients.

### 2.3 Outcomes and assessments

The primary endpoint used in this study was 2L-OS, which was defined as the time from the initiation of 2L therapy until the date of death of the patient due to any cause or the last day of follow-up. The secondary endpoints were 2L progression-free survival (2L-PFS), 2L objective response rate (2L-ORR), 2L disease control rate (2L-DCR), and safety. The 2L-PFS in this study was defined as the time from the initiation of 2L therapy until disease progression or death due to any cause. Tumor response was assessed based on the Response Evaluation Criteria in Solid Tumors (RECIST v1.1). The best overall response categories included complete response (CR), partial response (PR), stable disease (SD), and PD. The ORR in this study was defined as the proportion of patients who achieved CR or PR. DCR was defined as the proportion of patients who achieved CR, PR, or SD. Treatment-related adverse events (TRAEs) were graded based on the Common Terminology Criteria for Adverse Events version 5.0 (CTCAE v5.0).

### 2.4 Statistical analysis

In the primary analysis, patients with any missing data in the variables of interest were excluded, constituting a complete-case analysis. To evaluate the potential impact of missing data on our findings, we performed a sensitivity analysis using multiple imputation. Baseline characteristics for the categorical variables were presented as frequencies and percentages. Chi-squared or Fisher’s exact tests were performed to compare the categorical variables between the two groups. In order to minimize potential confounding factors, a propensity score matching (PSM) analysis was performed. The propensity scores were estimated using a logistic regression model that included all pre-specified baseline characteristics. One-to-one nearest-neighbor matching without replacement was then performed, utilizing a caliper width set to 0.02 standard deviations of the logit of the propensity score. This stringent caliper was applied to ensure that matched pairs were highly comparable. To quantitatively assess the balance of covariates between the matched groups, standardized mean differences (SMD) was calculated for all baseline variables. A successful balance was defined as an SMD of less than 0.1 for all key covariates following matching. The Kaplan-Meier method was adopted to estimate 2L-PFS and 2L-OS, and the differences between the two groups were assessed using the log-rank test. Univariate and multivariate Cox proportional hazards regression models were utilized to identify the factors associated with survival outcomes. The variables with a P-value of <0.15 in the univariate analysis were included in the multivariate model. The hazard ratio (HR) was reported with a 95% confidence interval (CI). Prespecified subgroup analyses (Gender [male or female], Age [≥ 65 or < 65 years], Smoking status [Yes or no], ECOG PS [≥ 2 or < 2], BMI [< 18.5, 18.5–24.9, 25.0–29.9 or ≥ 30.0], Lung metastasis [Yes or no], Bone metastasis [Yes or no]), Brain metastasis [Yes or no], Liver metastasis [Yes or no], Number of metastatic lesions [≥ 3 or < 3], 1L-PFS [≥ 6 or < 6 months] for 2L-PFS and 2L-OS were performed to assess the consistency of treatment effects in patient subgroups. Subgroup analyses employed an unstratified Cox proportional hazards model, with ICIs continuation status used as a covariate. A two-sided P-value < 0.05 was considered statistically significant. All the statistical analyses were conducted using SPSS version 25.0 (IBM Corp., Armonk, NY, USA).

## 3 Results

### 3.1 Baseline characteristics

Eligibility for the study required the availability of a complete medical record. Consequently, of the initially screened 216 patients, 5 were excluded due to incomplete data (3 lacking definitive 1L-PFS duration and 2 lacking clear documentation of the number of metastatic lesions.). A total of 211 patients were included in this study, among whom 118 formed the ICIs continuation group and 93 formed the ICIs discontinuation group. The median age of the included patients was 62 years (range: 19–87 years), and 81.5% of all patients were male, while 73% had a history of smoking. An ECOG PS ≥2 was present in 22.7% of patients. The majority of patients (63.0%) had normal weight (BMI 18.5-24.9); 29.9% were overweight (BMI 25.0-29.9). Metastatic involvement included lung (66.4% of the included cases), bone (32.7%), brain (21.3%), and liver (26.1%) involvement. Moreover, 76.3% of the patients presented with ≥3 metastatic lesions, whereas 60.2% of the patients achieved 1L-PFS ≥ 6 months. After PSM, the baseline characteristics were well balanced between the groups (**Table 1**).

**Table 1:** Baseline demographic and clinical characteristics of the patients.

Characteristics	Before PSM		p	After PSM		p
	ICIs continuation (n = 118)	ICIs discontinuation (n = 93)		ICIs continuation (n = 75)	ICIs discontinuation (n = 75)	
	No. (%)	No. (%)		No. (%)	No. (%)	
Gender			0.173			0.511
Male	100 (84.7)	72 (77.4)		64 (85.3)	61 (81.3)	
Female	18 (15.3)	21 (22.6)		11 (14.7)	14 (18.7)	
Age			0.332			0.611
≥65	47 (39.8)	31 (33.3)		26 (34.7)	29 (38.7)	
<65	71 (60.2)	62 (66.7)		49 (65.3)	46 (61.3)	
Smoking status			0.558			0.707
Yes	88 (74.6)	66 (71.0)		57 (76.0)	55 (73.3)	
No	30 (25.4)	27 (29.0)		18 (24.0)	20 (26.7)	
ECOG PS			0.780			0.554
≥2	26 (22.0)	22 (23.7)		18 (24.0)	15 (20.0)	
<2	92 (78.0)	71 (76.3)		57 (76.0)	60 (80.0)	
BMI			0.532			0.844
< 18.5	5 (4.2)	2 (2.2)		1 (1.3)	0 (0.0)	
18.5–24.9	77 (65.3)	56 (60.2)		47 (62.7)	50 (66.7)	
25.0–29.9	33 (28.0)	30 (32.3)		25 (33.3)	24 (32.0)	
≥ 30.0	3 (2.5)	5 (5.3)		2 (2.7)	1 (1.3)	
Lung metastasis			0.277			0.496
Yes	82 (69.5)	58 (62.4)		50 (66.7)	46 (61.3)	
No	36 (30.5)	35 (37.6)		25 (33.3)	29 (38.7)	
Bone metastasis			0.058			1.000
Yes	45 (38.1)	24 (25.8)		24 (32.0)	24 (32.0)	
No	73 (61.9)	69 (74.2)		51 (68.0)	51 (68.0)	
Brain metastasis			0.048			0.836
Yes	31 (26.3)	14 (15.1)		15 (20.0)	14 (18.7)	
No	87 (73.7)	79 (84.9)		50 (80.0)	61 (81.3)	
Liver metastasis			0.939			0.847
Yes	31 (26.3)	24 (25.8)		18 (24.0)	17 (22.7)	
No	87 (73.7)	69 (74.2)		57 (76.0)	58 (77.3)	
Number of metastatic lesions			0.522			0.850
≥3	92 (78.0)	69 (74.2)		56 (74.7)	57 (76.0)	
<3	26 (22.0)	24 (25.8)		19 (25.3)	18 (24.0)	
1L-PFS (months)			0.090			0.621
≥6	77 (65.3)	50 (53.8)		41 (54.7)	44 (58.7)	
<6	41 (34.7)	43 (46.2)		34 (45.3)	31 (41.3)	

### 3.2 Survival endpoints

**Figure 1:**
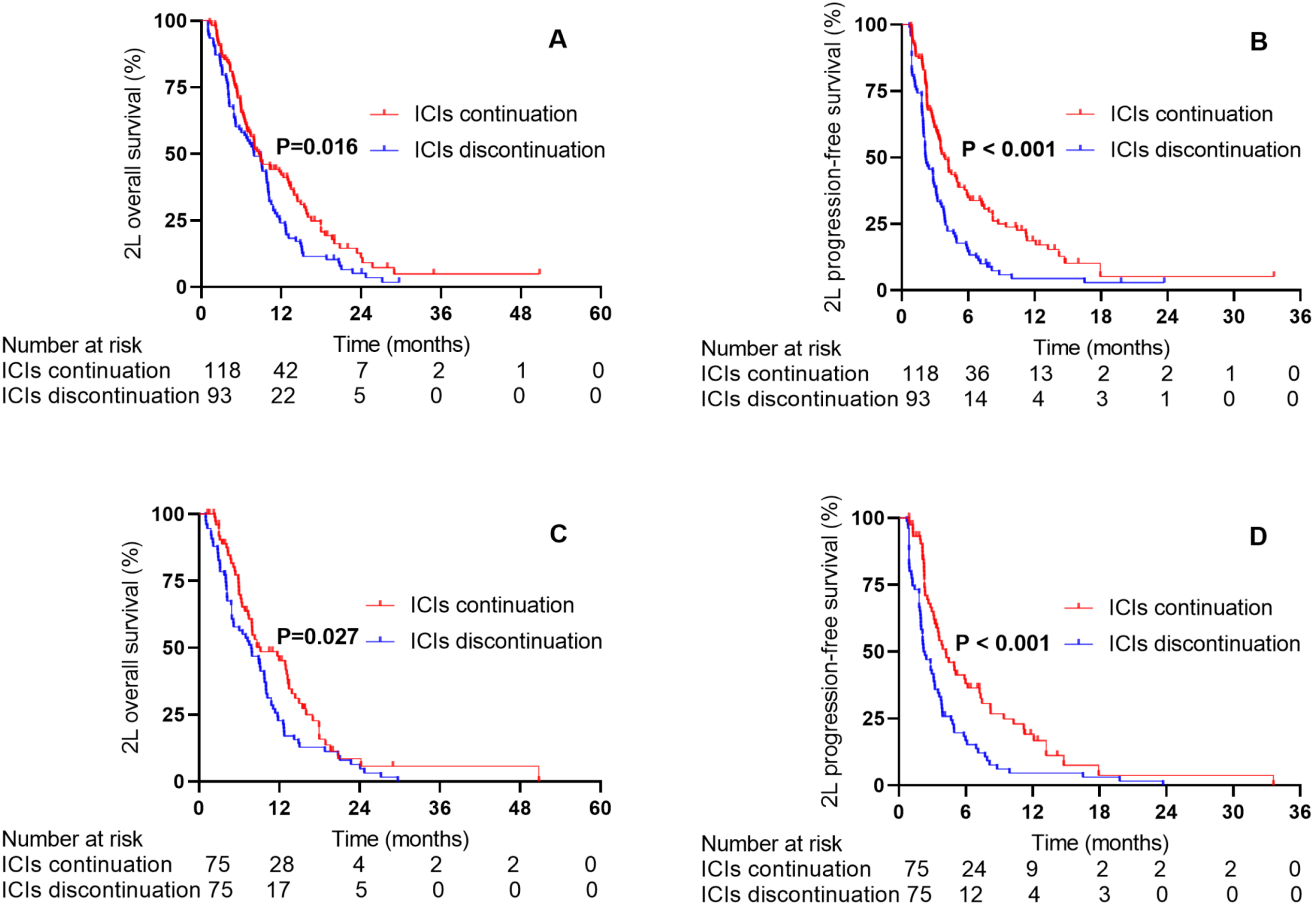
The 2L-OS and 2L-PFS between the ICIs continuation group and the ICIs discontinuation group before and after PSM. **(A)** 2L-OS before PSM. **(B)** 2L-PFS before PSM. **(C)** 2L-OS after PSM. **(D)** 2L-PFS after PSM.

In the entire study population, the median follow-up time was 18.90 months, and the median 2L-OS and 2L-PFS were 8.66 months (95% CI: 7.50–9.82) and 3.11 months (95% CI: 2.68–3.54), respectively. According to the 2L ICIs continuation status, the median 2L-OS was 8.66 months (95% CI: 6.66–10.66) in the ICIs continuation group and 7.90 months (95% CI: 5.78–10.02) in the ICIs discontinuation group (HR 0.70, 95% CI 0.52-0.94; P = 0.016, **Figure 1A**). The median 2L-PFS was 3.92 (3.28–4.56) in the ICIs continuation group and 2.15 (1.52–2.78) in the ICIs discontinuation group (HR 0.53, 95% CI 0.39-0.73; P < 0.001, **Figure 1B**).

After 1:1 PSM, the median 2L-OS and 2L-PFS were 9.08 months (95% CI: 7.43–10.73) and 3.11 months (95% CI: 2.60–3.63), respectively, in the entire cohort. The median 2L-OS was 10.31 months (95% CI: 6.49–14.13) in the ICIs continuation group and 8.95 months (95% CI: 5.60–12.30) in the ICIs discontinuation group (HR 0.67, 95% CI 0.48-0.95; P = 0.027, **Figure 1C**). The median 2L PFS was 4.22 months (95% CI: 2.75–5.69) in the ICIs continuation group and 2.12 months (95% CI: 1.63–2.61) in the ICIs discontinuation group (HR 0.52, 95% CI 0.37-0.74; P < 0.001, **Figure 1D**).

### 3.3 Tumor response

The 2L-ORR was 28.8% in the ICIs continuation group and 11.8% in the ICIs discontinuation group (p = 0.003), while the 2L-DCRs for the two groups were 65.3% and 44.1% (P = 0.002), **r**espectively. After 1:1 PSM, the 2L-ORR was 32.0% in the ICIs continuation group and 10.7% in the ICIs discontinuation group (P = 0.001), while the 2L-DCRs for the two groups were 65.3% and 42.7% (P = 0.005), **r**espectively (**Table 2**).

**Table 2:** Responses of second-line therapy.

Response	Before PSM		p	After PSM		p
	ICIs continuation (n = 118)	ICIs discontinuation (n = 93)		ICIs continuation (n = 75)	ICIs discontinuation (n = 75)	
	No. (%)	No. (%)		No. (%)	No. (%)	
CR	0	0		0	0	
PR	34 (28.8)	11 (11.8)		24 (32.0)	8 (10.7)	
SD	43 (36.4)	30 (32.3)		25 (33.3)	24 (32.0)	
PD	41 (34.7)	52 (55.9)		26 (34.7)	43 (57.3)	
2L-ORR	34 (28.8)	11 (11.8)	0.003	24 (32.0)	8 (10.7)	0.001
2L-DCR	77 (65.3)	41 (44.1)	0.002	49 (65.3)	32 (42.7)	0.005

### 3.4 Subgroup analysis

**Figure 2:**
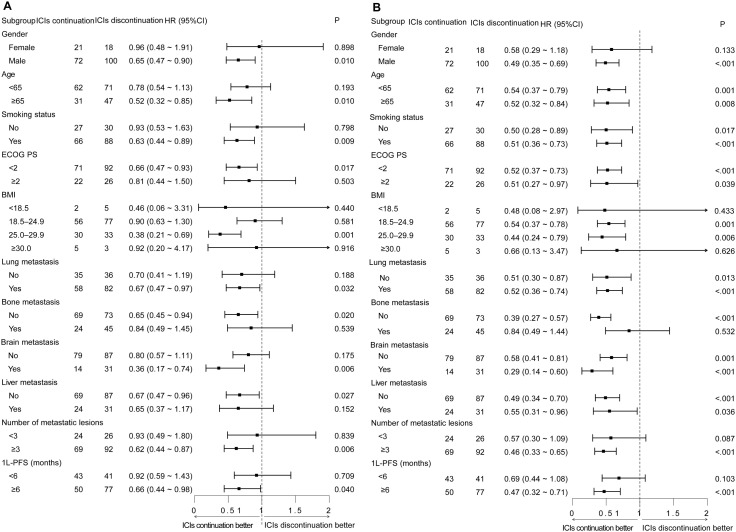
Subgroup analysis for 2L-OS and 2L-PFS in the entire population. **(A)** Subgroup analysis for 2L-OS in the entire population. **(B)** Subgroup analysis for 2L-PFS in the entire population.

Prespecified subgroup analyses stratified by baseline characteristics were performed. It was revealed that in most subgroups, ICIs continuation was beneficial in terms of 2L-OS and 2L-PFS (**Figures 2A, B**).

### 3.5 Safety

As shown in **Table 3**, except for the incidence of immune-related adverse events (irAEs), the incidence of treatment-related adverse events (TRAEs) was similar between the two groups, and no grade 4 or 5 AEs were noted. The observed irAEs included hypothyroidism, rash, pneumonitis, diarrhea, and adrenal insufficiency. Only one patient (0.5%) developed a grade 3 immune-mediated rash, while no grade 4 or 5 irAEs were recorded.

**Table 3:** TRAEs for the two groups of patients.

TRAEs	ICIs continuation (n = 118)		ICIs discontinuation (n = 93)		P
	n	%	n	%	
Hematologic toxicities	72	61.0	56	60.2	0.906
G3/4 hematologic toxicities	21	17.8	17	18.3	0.928
Gastrointestinal toxicities	56	47.5	43	46.2	0.860
G3/4 gastrointestinal toxicities	12	10.2	10	10.8	0.891
Hepatic toxicities	44	37.3	33	35.5	0.787
G3/4 elevated ALT/AST	7	5.9	6	6.5	0.876
Hypothyroidism	9	7.6	0	0	0.005
Rash	8	6.8	0	0	0.010
G3 rash	1	0.5	0	0	1.000
Pneumonitis	2	2.0	0	0	0.505
Diarrhea	1	0.8	0	0	1.000
Adrenal insufficiency	2	1.7	0	0	0.505

### 3.6 Cox regression analysis for 2L-PFS and 2L-OS

In the ICIs continuation group, the risk factors affecting 2L-OS and 2L-PFS were explored next in this study. Multivariate Cox regression analyses revealed baseline liver metastasis as an independent factor associated with worse 2L-OS and 2L-PFS and 1L-PFS as an independent factor associated with favorable 2L-OS and 2L-PFS. Additionally, overweight (BMI 25.0-29.9) was identified as an independent prognostic factor for favorable 2L-OS (**Tables 4**, **5**).

**Table 4:** Univariate and multivariate Cox regression analyses for 2L-OS.

Characteristics	Univariate analysis		Multivariate analysis	
	HR, 95%CI	P	HR, 95%CI	P
Gender	0.84 (0.48–1.48)	0.554		
Male				
Female				
Age	1.18(0.77–1.81)	0.439		
≥65				
<65				
Smoking status	0.82 (0.52–1.30)	0.401		
Yes				
No				
ECOG PS	1.14(0.70–1.86)	0.606		
≥2				
<2				
BMI				
< 18.5	0.54 (0.20–1.50)	0.240	0.60 (0.21–1.70)	0.332
18.5–24.9	–	–	–	–
25.0–29.9	0.52 (0.31–0.87)	0.012	0.57 (0.34–0.97)	0.039
≥ 30.0	0.98 (0.31–3.13)	0.972	0.75 (0.23–2.48)	0.634
Lung metastasis	1.37(0.87–2.18)	0.178		
Yes				
No				
Bone metastasis	1.07(0.70–1.64)	0.751		
Yes				
No				
Brain metastasis	0.82 (0.51–1.33)	0.427		
Yes				
No				
Liver metastasis	2.02(1.30–3.16)	0.002	1.96 (1.22–3.14)	0.005
Yes				
No				
Number of metastatic lesions	1.45(0.85–2.47)	0.171		
≥3				
<3				
1L-PFS (months)	0.45(0.30–0.69)	<0.001	0.58 (0.37–0.91)	0.018
≥6				
<6				

**Table 5:** Univariate and multivariate Cox regression analyses for 2L-PFS.

Characteristics	Univariate analysis		Multivariate analysis	
	HR, 95%CI	P	HR, 95%CI	P
Gender	0.85 (0.49–1.49)	0.579		
Male				
Female				
Age	1.14 (0.74–1.74)	0.551		
≥65				
<65				
Smoking status	0.96 (0.60–1.52)	0.859		
Yes				
No				
ECOG PS	1.15 (0.70–1.87)	0.580		
≥2				
<2				
BMI				
< 18.5	0.90 (0.33–2.48)	0.841	0.93 (0.33–2.63)	0.893
18.5–24.9	–	–	–	–
25.0–29.9	0.60 (0.36–0.99)	0.045	0.72 (0.43–1.21)	0.215
≥ 30.0	1.16 (0.36–3.70)	0.808	0.88 (0.26–3.01)	0.836
Lung metastasis	1.40 (0.88–2.22)	0.156		
Yes				
No				
Bone metastasis	1.21 (0.79–1.85)	0.375		
Yes				
No				
Brain metastasis	0.87 (0.54–1.41)	0.581		
Yes				
No				
Liver metastasis	2.26 (1.44–3.54)	<0.001	1.88 (1.15–3.08)	0.012
Yes				
No				
Number of metastatic lesions	1.83 (1.08–3.10)	0.025	1.18 (0.65–2.14)	0.582
≥3				
<3				
1L-PFS (months)	0.45 (0.30–0.68)	<0.001	0.55 (0.35–0.89)	0.014
≥6				
<6				

### 3.7 Continuing ICIs vs. switching the ICIs

In the ICIs continuation cohort, 105 patients received the same ICIs throughout the 2L therapy, and this group was defined as the continuing ICIs group. The remaining 13 patients who received 2L ICIs therapy with treatment switched between anti-PD-1 and anti-PD-L1 antibodies were designated as the switching ICIs group. The baseline characteristics of the two groups were essentially the same (**Table 6**).

**Table 6:** Baseline characteristics between the continuing ICIs group and the switching ICIs group.

Characteristics	Continuing ICIs (n = 105)		Switching ICIs (n = 13)		P
	No	(%)	No	(%)	
Gender					1.000
Male	89	84.8	11	84.6	
Female	16	15.2	2	15.4	
Age					0.191
≥65	44	41.9	3	23.1	
<65	61	58.1	10	76.9	
Smoking status					0.091
Yes	81	77.1	7	53.8	
No	24	22.9	6	46.2	
ECOG PS					0.293
≥2	25	23.8	1	7.7	
<2	80	76.2	12	92.3	
BMI					1.000
< 18.5	5	4.8	0	0.0	
18.5–24.9	68	64.8	9	69.2	
25.0–29.9	29	27.6	4	30.8	
≥ 30.0	3	2.9	0	0.0	
Lung metastasis					1.000
Yes	73	69.5	9	69.2	
No	32	30.5	4	30.8	
Bone metastasis					0.365
Yes	42	40.0	3	23.1	
No	63	60.0	10	76.9	
Brain metastasis					1.000
Yes	28	26.7	3	23.1	
No	77	73.7	10	76.9	
Liver metastasis					0.180
Yes	30	28.6	1	7.7	
No	75	71.4	12	92.3	
Number of metastatic lesions					0.479
≥3	83	79.0	9	69.2	
<3	22	21.0	4	30.8	
1L-PFS (months)					0.032
≥6	65	61.9	12	92.3	
<6	40	38.1	1	7.7	

**Figure 3:**
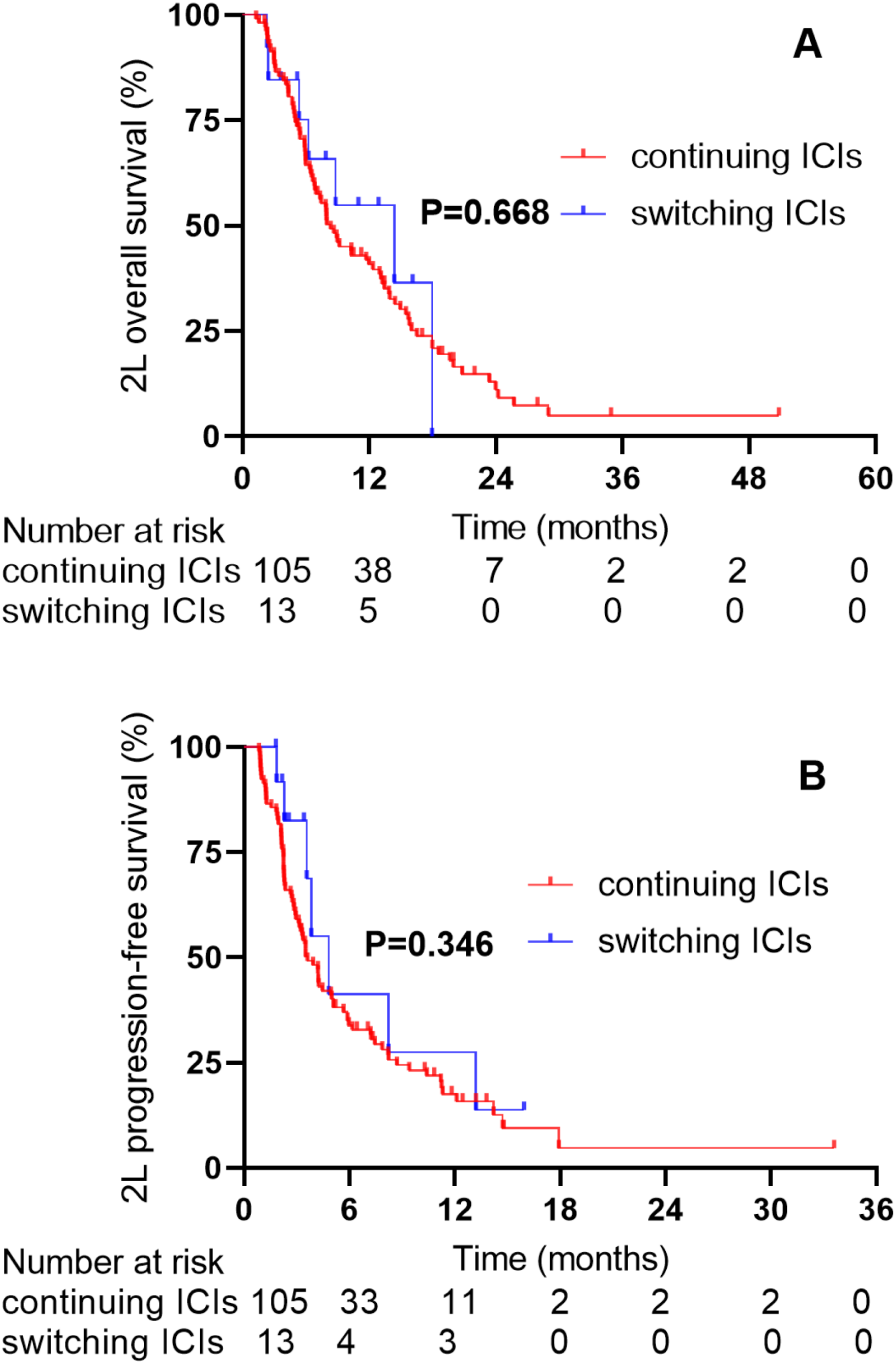
The 2L-OS and 2L-PFS between the continuing ICIs group and the switching ICIs group. **(A)** 2L-OS for the continuing ICIs group and the switching ICIs group. **(B)** 2L-PFS for the continuing ICIs group and the switching ICIs group.

The median follow-up time was 16.10 months in ICIs continuation cohort. The median 2L-OS was 8.36 months (95% CI: 5.89–10.83) in the continuing ICIs group and 14.37 months (95% CI: 4.50–24.24) in the switching ICIs group (HR 1.18, 95% CI 0.58-2.43; P = 0.668, **Figure 3A**). In these two groups, the median 2L-PFS was 3.60 months (95% CI: 2.78–4.42) and 4.83 months (95% CI: 2.27–7.39), respectively (HR 1.44, 95% CI 0.74-2.80; P = 0.346, **Figure 3B**). The incidence of TRAEs was comparable between these two groups.

## 4 Discussion

ICIs have significantly reshaped the landscape of ES-SCLC treatment, especially in 1L therapy. The landmark trial IMpower133, CASPIAN established chemoimmunotherapy as the standard treatment in 1L settings, achieving improved median OS compared to that of patients receiving chemotherapy alone (8, 9). Unfortunately, an overwhelmingly large proportion of patients eventually experience disease progression, within months, due to which they have to undergo therapeutic management in 2L treatment settings, which is a phase with profound clinical challenges due to a paucity of effective, well-tolerated options (topotecan, lurbinectedin offering only modest benefits), rapidly declining performance status, and devastatingly short median survival, often measured in weeks. This has driven the exploration of effective 2L therapeutic approaches that could prolong survival and also improve the quality of life of ES-SCLC patients who experience disease progression after 1L chemoimmunotherapy, and this exploration includes investigating the efficacy of the continuation of ICIs for such patients in 2L treatment settings.

The present study represents one of the largest and most comprehensive investigations of the efficacy of ICIs continuation in the 2L treatment of ES-SCLC after disease progression is observed after 1L chemoimmunotherapy. The large sample size and multicenter design of this study provide robust data, which increases the generalizability of the findings. The inclusion of patients from multiple centers facilitated capturing a diverse patient population, which could reflect the real-world clinical settings accurately. This approach also minimizes the potential selection bias and ensures that the results are applicable to a broader range of patients with ES-SCLC.

The findings of this study demonstrated that ICIs continuation in 2L therapy settings is associated with substantial improvements in 2L-OS and 2L-PFS. Specifically, the patients with continued ICIs therapy in 2L settings presented significantly greater 2L-OS and 2L-PFS than those of patients with discontinuation of ICIs. These findings are consistent with the hypothesis that prolonged immunotherapy can maintain the immune control of the disease, leading to better clinical outcomes in patients. The observed improvements in 2L-OS and 2L-PFS suggest that the continuation of ICIs in 2L settings can effectively delay disease progression and prolong survival, indicating this as a valuable therapeutic option for patients who exhibit disease progression after 1L chemoimmunotherapy. In addition to the survival benefits, higher 2L-ORRs and 2L-DCRs were noted in the ICIs continuation group, indicating that continuation of ICIs therapy in 2L settings not only prolongs survival but also enhances tumor control, potentially leading to a better quality of life for the patients. The safety profile of patients when continuing ICIs was found to be manageable. In our study, most irAEs were low-grade and were successfully managed with supportive care. The most frequently observed irAE was hypothyroidism, which occurred in nine patients and was managed either with close monitoring or thyroid hormone replacement therapy. Only one patient (0.5%) developed a grade 3 immune-mediated rash; this event resolved after a brief delay in ICI dosing and the administration of systemic corticosteroids. Overall, these all suggest that long-term ICI therapy was well-tolerated in our cohort, with a manageable safety profile and no observed increase in the incidence of irAEs. The manageable safety profile observed in this study supports the notion that ICIs continuation can be safely administered in 2L settings, serving as an alternative treatment strategy for patients with ES-SCLC.

The mechanism underlying the efficacy of ICIs continuation in the 2L setting could be attributed to the unique immunological landscape of ES-SCLC. SCLC is characterized by a high mutational burden and expression of immune checkpoint molecules, such as PD-L1, rendering ICIs therapy suitable for these patients. ICIs continuation may help maintain the ability of the immune system to recognize and attack cancer cells, thereby prolonging the therapeutic effect for the patients. Additionally, the combination of ICIs with chemotherapy in the 1L setting prepares the immune system for a more responsive activity upon ICIs continuation in 2L settings.

In our study, the observed 1.36-month improvement in median 2L-OS corresponds to a 33% reduction in the risk of death, a finding considered clinically meaningful given the poor prognosis of second-line ES-SCLC. Furthermore, the strategy’s favorable safety profile and potential to enable long-term survival for a subset of patients support its relevance in real-world practice. However, the modest survival benefit also highlight the ongoing challenge of immunotherapy resistance in ES-SCLC. To address this limitation, organoid technology emerges as a powerful platform for investigating immune resistance mechanisms. By recapitulating the tumor microenvironment (TME), preserving tumor heterogeneity, and enabling drug screening, mechanistic studies, and personalized immune co-culture models, organoids help identify resistance pathways and inform therapeutic development (26). Within the TME, senescent macrophages promote tumor progression and immune evasion through the senescence-associated secretory phenotype (SASP), which secretes immunosuppressive cytokines and inflammatory factors. Targeting the SASP may restore macrophage function and enhance antitumor immunity (27). Concurrently, exosome-related proteomic and glycoproteomic studies have established glycosylation as a key regulator of lung cancer progression, providing a rationale for glycoprotein-targeted agents to overcome treatment resistance (28). However, clinical translation will require broader validation and improved glycoprotein enrichment techniques. In summary, overcoming immunotherapy resistance in ES-SCLC demands a multi-pronged strategy, integrating advanced models such as organoids to decipher mechanisms, alongside novel therapies targeting immune-suppressive elements like senescent macrophages and tumor-specific modifications such as glycoproteins. Such an integrated approach is essential to break through the current therapeutic plateau and achieve meaningful clinical advances.

The benefits of ICIs continuation were also observed across the different subgroups analyzed in this study, including the patients with different age groups, performance statuses, and metastatic sites. The findings of the subgroup analysis suggested that continued ICI therapy may be effective for a broad range of patients with ES-SCLC, regardless of their specific clinical characteristics. This finding is particularly significant, as it highlights the potential applicability of this treatment strategy in diverse patient populations. However, it is important to acknowledge that some subgroups are characterized by small sample sizes, resulting in wide confidence intervals. This suggests a degree of uncertainty in the estimates for these smaller subgroups. Therefore, these findings should be interpreted with caution.

Next, to elucidate the factors influencing outcomes in this specific population, a multivariable Cox proportional hazards analysis was performed. The analysis revealed that both liver metastasis and 1L-PFS were independent prognostic factors for 2L-OS and 2L-PFS in patients who continued the ICIs therapy. The presence of liver metastasis is a well-recognized factor indicating poor prognosis in many types of cancer, including SCLC (29, 30). Liver metastasis often indicates more aggressive disease biology and a greater tumor burden, which may contribute to the poorer outcomes observed in these patients (30–32). Liver metastasis may also affect the overall health status of patients, leading to a reduced tolerance to treatment and a greater likelihood of treatment-related complications. Some studies have shown that SCLC patients with liver metastasis benefit less from ICIs treatment than patients without liver metastasis (32). In this study, patients with liver metastasis exhibited evidently poorer prognosis, indicating that liver metastasis could be a useful marker for the identification of high-risk patients who may require more aggressive treatment strategies. Conversely, a longer 1L PFS interval was revealed as a strong indicator of favorable prognosis in 2L settings and likely reflects the inherent tumor biology, treatment sensitivity, and potentially a more favorable immune context. Therefore, 1L-PFS serves as a valuable clinical marker for risk stratification after disease progression. This finding underscores the importance of optimizing 1L treatment to achieve extended disease control, as patients with shorter 1L-PFS may benefit from more intensive 2L therapies. The significance of 1L-PFS as a prognostic factor revealed in this study highlights the importance of achieving durable responses with 1L therapy. Patients who experience rapid disease progression during 1L treatment might have more aggressive disease biology and may, therefore, be less responsive to subsequent therapies. This finding suggests that optimizing 1L treatment strategies, for example by using combination therapies or novel agents, may be crucial for improving the long-term outcomes for patients with ES-SCLC. Furthermore, our analysis identifies overweight (BMI 25-29.9) as an independent prognostic factor associated with superior 2L-OS in patients who continued the ICIs therapy. This finding aligns with the “obesity paradox” previously observed in lung cancer, wherein an elevated BMI is correlated with superior survival outcomes (33–35) and a better immunologic response (34) in patients who received ICIs. Potential mechanisms include enhanced metabolic reserves counteracting cancer cachexia and immunometabolic interactions that potentiate treatment response (36). Therefore, our results reinforce the value of baseline BMI as a practical clinical indicator for risk stratification and outcome prediction in patients receiving ICIs therapy.

Research on the efficacy of switching between anti-PD-1 and anti-PD-L1 antibodies as 2L therapy in ES-SCLC remains limited currently. Switching between the administration of anti-PD-1 and anti-PD-L1 antibodies is considered an effective and safe treatment option for certain selected advanced or recurrent patients (37, 38). Therefore, in this study, the impact of switching ICIs in 2L settings was investigated for ES-SCLC patients. Interestingly, no significant differences in 2L-PFS and 2L-OS were revealed between patients who continued receiving the same ICIs and those who were switched between anti-PD-1 and anti-PD-L1 antibodies. However, a trend toward prolonged 2L-OS and 2L-PFS was noted in the switching ICIs group compared to the continuing ICIs group. This is consistent with the findings of Liu et al. (22). The lack of significant differences in the effects between continued and switching ICIs for patients in 2L settings could be attributed to the relatively small sample size in the switching ICIs cohort in this study, which might have limited the statistical power of the analysis. Additionally, the biological rationale for switching remains unclear to date, and further studies are needed to elucidate the potential mechanisms and benefits of such a strategy. Importantly, the decision to switch ICIs should be carefully considered in clinical practice. While this study did not reveal significant differences in the outcomes, the potential benefits of switching ICIs may vary depending on individual patient characteristics and the specific ICIs used. Future prospective studies with larger cohorts and longer follow-up periods are, therefore, warranted to provide more definitive answers regarding the efficacy and safety of switching ICIs in the 2L treatment of patients with ES-SCLC. The lack of significant differences in the outcomes between the continuing ICIs and switching ICIs cohorts may also reflect the complex interplay between the immune system and cancer cells. The efficacy of ICIs is influenced by several factors, including the expression of immune checkpoint molecules, the presence of immune-infiltrating cells in the tumor microenvironment, and the overall immune status of the patient (38–40). Therefore, switching the administration of anti-PD-1 and anti-PD-L1 antibodies may not necessarily provide additional benefits to patients if the underlying immune mechanisms are not significantly different.

This study highlighted the safety and efficacy of ICIs continuation for patients who experience disease progression after 1L chemoimmunotherapy. The findings of this study suggest that ICIs continuation can be a viable treatment option for such patients and may serve as an alternative strategy in clinical practice. However, it is important to recognize that the survival benefits observed in this study were limited, warranting further research to develop novel therapeutic agents that would enable achieving improved outcomes for patients with ES-SCLC. Moreover, novel combination therapies, including integrated targeted therapies, novel immunotherapies, and other emerging treatments, hold promise in terms of further improving the prognosis of patients with ES-SCLC (15, 41–43). The identification of predictive biomarkers that can further assist in identifying patients who are most likely to benefit from ICI continuation or other therapeutic strategies is, therefore, a critical future research direction. Prospective studies with well-defined patient cohorts and robust biomarker analyses are, therefore, needed to advance the current understanding of the optimal treatment approaches for ES-SCLC. One potential avenue for improving outcomes in patients with ES-SCLC is the development of combination therapies targeting multiple aspects of disease biology. For example, the combined use of ICIs with targeted therapies that inhibit specific oncogenic pathways, such as the PI3K/AKT/mTOR pathway, could enhance the therapeutic effect by simultaneously targeting the immune system and cancer cells (44). In addition, emerging treatments such as CAR-T-cell therapy, oncolytic viruses, and novel checkpoint inhibitors targeting other immune checkpoints may be opted for patients who do not respond to the standard ICIs (6, 45–47). Another important research area could be the identification of predictive biomarkers that would facilitate the customization of treatment strategies to individual patients. However, to date, there has been no breakthrough in the research exploring effective biomarkers that could enable the selection of patients at an advantage of immunotherapy. While PD-L1 expression has been used as a biomarker for ICIs in other cancers (48), its utility in ES-SCLC remains to be elucidated. Future studies should focus on identifying additional biomarkers, which could include tumor mutational burden, immune cell infiltrates, and circulating tumor DNA, for better predicting the response to ICIs and other therapies (49, 50). Personalized treatment approaches based on these biomarkers can improve the efficacy and safety of therapies for patients with ES-SCLC.

It is important to acknowledge that, while this study provides valuable insights into the efficacy and safety of ICIs continuation in the 2L treatment of ES-SCLC, it also has certain limitations. First, the retrospective and non-randomized design is susceptible to selection bias and precludes definitive causal inference. Additionally, the potential for incomplete or inconsistent data collection may affect the accuracy of the results. Second, heterogeneity in patient populations and treatment protocols across participating centers could have influenced outcome assessments. A key concern is the potential for unmeasured confounders—such as socioeconomic status, specific comorbidities, ICI types, or unassessed molecular features—which, despite adjustment for known prognostic factors, may bias treatment effect estimates and compromise the internal validity of our findings. Finally, the analysis of the switching ICIs cohort was likely underpowered due to its small sample size. Future prospective studies, ideally randomized controlled trials, are warranted to validate these results.

In conclusion, this study demonstrated that ICIs continuation in the 2L treatment of patients with ES-SCLC is associated with improved survival outcomes and a manageable safety profile. ICIs continuation can, therefore, be considered a viable treatment option for patients who experience disease progression after 1L chemoimmunotherapy.

## Data Availability

The raw data supporting the conclusions of this article will be made available by the authors, without undue reservation.
